# Management of spontaneous septic hypothermia in intensive care. A national survey of French intensive care units

**DOI:** 10.3389/fmed.2024.1393781

**Published:** 2024-06-06

**Authors:** Gabriel Eustache, Pierre Le Balc’h, Yoann Launey

**Affiliations:** Critical Care Unit, Department of Anaesthesia, Critical Care and Perioperative Medicine, University Hospital of Rennes, Rennes, France

**Keywords:** spontaneous septic hypothermia, sepsis, septic shock, temperature control, hypothermia

## Abstract

The benefit of temperature control in sepsis or septic shock is still under debate in the literature. We developed a national survey to assess the current state of knowledge and the practical management of spontaneous septic hypothermia in French intensive care units. Out of more 764 intensivists who were contacted, 436 responded to the survey. The majority of doctors (52.4%) considered spontaneous septic hypothermia to be a frequently encountered situation in intensive care, and 62.1% were interested in this problem. Definition of spontaneous septic hypothermia among French intensivists was not consensual. More than half of the doctors questioned (57.1%) stated that they did not actively rewarm patients suffering from spontaneous septic hypothermia.

## Introduction

The benefit of temperature control in sepsis or septic shock is still under debate in the literature (1, 2). The concept of a protective and adaptive effect of fever is controversial (3, 4) and the current randomised trial, SEPSISCOOL 2 (NCT04494074), compares two thermal control strategies for febrile patients in septic shock undergoing mechanical ventilation: namely, maintaining fever and maintaining normothermia via external cooling. However, a recent pilot study of afebrile septic patients found all-cause mortality at 28 days to be lower when hyperthermia (increase in body temperature of +1.5°C) was induced by external rewarming (5). In contrast, spontaneous hypothermia is thought to be associated with increased mortality among patients with sepsis (6). The benefits of induced hypothermia have also been reported in animal studies (7, 8), but clinical benefits have not been demonstrated in mechanically ventilated human patients with septic shock (9).

Unlike accidental hypothermia (10), hypothermia associated with haemorrhagic shock (11), or perioperative-associated hypothermia (12), there is no consensus on the management of spontaneous septic hypothermia. Two surveys—one involving patients in the United Kingdom (13) and the other on a European scale (14)—looked at the practices of different intensivists with regards to hypothermia in septic patients. Both studies revealed great variability in the definition and clinical management of the condition.

The aim of this survey is to assess the current state of knowledge and the practical management of spontaneous septic hypothermia in French intensive care units.

## Methods

We developed a national survey containing simple or multiple-choice questions and open questions. In the first phase, the survey was distributed to intensivists in the surgical intensive care unit at Rennes University Hospital. In the second phase, the survey was submitted to doctors working in intensive care units in other departments (medical and cardio-thoracic intensive care) for testing and validation. The questions were revised and adapted according to the comments received. It was then circulated to members of the SFAR (French Society of Anesthesia and Intensive Care Medicine) and SRLF (French Intensive Care Society) societies between 1 March and 4 July 2023. Respondents were asked to answer the survey anonymously, referring to the usual practices within their respective intensive care units. The survey was distributed by e-mail to SRLF members, and also distributed to SFAR members including through social networks.

## Statistical analysis

All analyses and graphs were produced using Excel® software. Categorical variables were presented as counts and percentages.

## Results

The survey was distributed by e-mail (764 e-mails) and through social networks. However, we do not know exactly how many intensivists actually received the survey and so cannot calculate an accurate response rate. The 2021 French demographic survey reported 2,350 intensivists practicing in France (15), of whom we hope to have contacted close to 50%.

Out of more 764 intensivists who were contacted, 436 responded to the survey. Of these, 405 worked in public hospitals, almost half in general intensive care units. The most represented specialisation was anaesthesiology. Over one-third of respondents declared 0–5 years’ experience in intensive care. All French regions were represented, as were the majority of departments (96%). Table 1 presents the characteristics of survey respondents.

**Table 1:** Characteristics of respondents.

Characteristics	All respondents (*n* = 436)
Intensive care, *n* (%)	
Polyvalent	215 (49.3)
Medical	117 (26.8)
Surgical	100 (22.9)
Polyvalent	58 (59.8)
Neurosurgery-neurology	16 (16.5)
Cardiothoracic	23 (23.7)
Paediatrics	4 (0.9)
Hospital, *n* (%)	
Public	405 (92.9)
Academic	262 (60.1)
Nonacademic	143 (32.8)
Private	28 (6.4)
Armies	3 (0.7)
Years of experience, *n* (%)	
0–5 years	170 (39.1)
6–10 years	98 (22.5)
11–15 years	70 (16.1)
16–20 years	33 (7.6)
>20 years	64 (14.7)
Diploma in specialised studies, *n* (%)	
Anaesthesiology	238 (55.1)
Medical	114 (26.4)
Intensivist	52 (12)
Emergency medicine	28 (6.5)
Number of intensive care beds, *n* (%)	
<10	37 (8.5)
10–15	139 (32.1)
16–20	143 (33)
21–25	65 (15)
26–30	41 (9.5)
>30	8 (1.8)
Region, *n* (%)	
Auvergne-Rhône-Alpes	53 (12.3)
Bourgogne-Franche-Comté	12 (2.8)
Bretagne	67 (15.5)
Centre-Val de Loire	22 (5.1)
Corse	1 (0.2)
Grand Est	31 (7.2)
Hauts-de-France	15 (3.5)
Île-de-France	112 (25.9)
Normandie	32 (7.4)
Nouvelle-Aquitaine	16 (3.7)
Occitanie	17 (13.9)
Pays de la Loire	23 (5.3)
Provence-Alpes-Côte d’Azur	25 (5.8)
Outre-mer	6 (1.4)

The majority of doctors (52.4%) considered spontaneous septic hypothermia to be a frequently encountered situation in intensive care, and 62.1% were interested in this problem. Table 2 illustrates the heterogeneity in the definition of spontaneous septic hypothermia among French intensivists.

**Table 2:** The temperatures below which respondents consider a patient with sepsis hypothermic.

Definition of hypothermia (°C)	Response rate (*N* = 418)
33°C	1 (0.2%)
34°C	6 (1.4%)
35°C	107 (25.6%)
35.5°C	36 (8.6%)
35.8°C	4 (1.0%)
36°C	241 (57.7%)
36.3°C	2 (0.5%)
36.5°C	19 (4.5%)
37°C	2 (0.5%)

More than half of the doctors questioned (57.1%) stated that they did not actively rewarm patients suffering from spontaneous septic hypothermia, but 42.3% of these reported using survival blankets to limit heat loss. The primary reason for not using active rewarming (reported by 76.1% of respondents) was the lack of evidence in the literature and 30% of intensivists who did not use it actually associated the practice with deleterious effects, particularly in terms of haemodynamics. Furthermore, 30% of doctors who did not use active rewarming considered hypothermia to be an adaptive response that should be tolerated, and 5.2% thought it may have beneficial effects.

Of the doctors prescribing active rewarming, 80% used it in cases of sepsis or septic shock and 17% used it for septic shock only. The vast majority (97.3%) administered rewarming using pulsed hot-air blankets, while rewarming through infusion fluids and targeted temperature control equipment were rarely used (by 6.5 and 14%, respectively). Most of the doctors who used active rewarming reported starting it at 35°C (Table 3) with a target temperature of 36°C (Table 4). The speed at which hypothermia was corrected was uncontrolled by 52.4% of doctors, with 36.8 and 9.2% reportedly aiming for 0.5 and 1°C/h, respectively.

**Table 3:** The trigger temperature at which respondents consider rewarming patients with hypothermic sepsis.

Trigger for rewarming (°C)	Response rate (*N* = 183)
32°C	1 (0.5%)
33°C	1 (0.5%)
34°C	13 (7.1%)
34.5°C	4 (2.2%)
35°C	66 (36.1%)
35.5°C	50 (27.3%)
36°C	46 (25.1%)
36.5°C	2 (1.1%)

**Table 4:** The target temperature to which respondents rewarm patients.

Rewarming target temperature (°C)	Response rate (*N* = 189)
35°C	2 (1.1%)
36°C	88 (49.2%)
36.3°C	1 (0.6%)
36.5°C	37 (20.7%)
37°C	48 (26.8%)
37.5°C	3 (1.7%)

Regarding the reasons for practising active rewarming, the majority of respondents (69.8%) wanted to combat coagulation disorders induced by hypothermia. Half of the intensivists surveyed used this practice because of the excess mortality associated with septic hypothermia, and 41.2% implemented rewarming for the immunomodulatory effects. Among the other responses, clinical tolerance of hypothermia and shivering were cited by 3% as justification for rewarming, while the prevention of hypothermia-induced cardiovascular events was reported by 4%.

## Conclusion

There is currently no consensual definition of spontaneous septic hypothermia, and huge heterogeneity exists in the management of this condition with a poor prognosis. The results of the present survey provide an overview of the clinical practices in French intensive care units, which are mainly based on medical experience or extrapolation from the management of hypothermic non-septic patients (haemorrhagic shock, accidental hypothermia, peri-operative hypothermia). The data presented here highlight the gaps in the current literature on this subject. The publications are mainly descriptive or pathophysiological and do not make it possible to identify a clear and consensual definition or a strategy for the therapeutic management of spontaneous septic hypothermia. No therapeutic trial has assessed the impact of active rewarming in terms of morbidity and mortality. This could explain the results of our survey, in which less than half of the doctors questioned practised active rewarming, with the methods and objectives varying widely from centre to centre. Our findings highlight the necessity of further therapeutic trials involving intensive care teams for improving the management of patients with spontaneous septic hypothermia.

## Data availability statement

The original contributions presented in the study are included in the article/supplementary material, further inquiries can be directed to the corresponding author.

## Author contributions

GE: Conceptualization, Data curation, Formal analysis, Funding acquisition, Writing – original draft, Writing – review & editing. PB: Writing – review & editing. YL: Writing – review & editing.
